# Tear Film Alterations in Type 2 Diabetes Mellitus: A Systematic Review and Meta-Analysis

**DOI:** 10.3390/diagnostics15243104

**Published:** 2025-12-06

**Authors:** Delius Mario Ghenciu, Alexandra Ioana Dănilă, Emil Robert Stoicescu, Adrian Neagu, Laura Andreea Ghenciu

**Affiliations:** 1 Doctoral School, Victor Babeş University of Medicine and Pharmacy Timisoara, Eftimie Murgu Square No. 2, 300041 Timisoara, Romania; mario.ghenciu@umft.ro; 2 Department of Functional Sciences, Victor Babeş University of Medicine and Pharmacy Timisoara, Eftimie Murgu Square No. 2, 300041 Timisoara, Romania; bolintineanu.laura@umft.ro; 3 Center for Modeling Biological Systems and Data Analysis, Victor Babeş University of Medicine and Pharmacy Timisoara, Eftimie Murgu Square No. 2, 300041 Timisoara, Romania; 4 Department of Anatomy and Embryology, Victor Babeş University of Medicine and Pharmacy Timisoara, Eftimie Murgu Square No. 2, 300041 Timisoara, Romania; alexandra.danila@umft.ro; 5 Department of Radiology and Medical Imaging, Victor Babeş University of Medicine and Pharmacy Timisoara, Eftimie Murgu Square No. 2, 300041 Timisoara, Romania; stoicescu.emil@umft.ro; 6 Research Center for Pharmaco-Toxicological Evaluations, Victor Babeş University of Medicine and Pharmacy Timisoara, Eftimie Murgu Square No. 2, 300041 Timisoara, Romania; 7 Field of Applied Engineering Sciences, Specialization Statistical Methods and Techniques in Health and Clinical Research, Faculty of Mechanics, ‘Politehnica’ University Timisoara, Mihai Viteazul Boulevard No. 1, 300222 Timisoara, Romania; 8 Department of Physics and Astronomy, University of Missouri, Columbia, MO 65211, USA; 9 Center for Translational Research and Systems Medicine, Victor Babeş University of Medicine and Pharmacy Timisoara, Eftimie Murgu Square No. 2, 300041 Timisoara, Romania

**Keywords:** dry eye disease, Schirmer test, tear break-up time, tear meniscus height, ocular surface disease

## Abstract

**Background**: Type 2 diabetes mellitus (T2DM) is increasingly recognized as affecting not only the retina but also the ocular surface. Chronic hyperglycemia can disrupt meibomian gland function, reduce tear secretion, and impair corneal sensitivity, leading to tear film instability and symptoms of dry eye disease. However, previous studies have reported variable findings, and the extent of these alterations remains uncertain. **Methods**: Following PRISMA guidelines, this systematic review and meta-analysis evaluated observational studies that compared tear film parameters between adults with T2DM and non-diabetic controls. Eligible studies assessed one or more of the following: invasive or non-invasive tear break-up time, Schirmer test, tear meniscus height, or Ocular Surface Disease Index (OSDI). **Results**: Twenty-four studies involving approximately 3500 eyes were included. Most reported significantly reduced tear stability and secretion in diabetic participants compared with controls. Tear break-up times were consistently shorter in T2DM, indicating a less stable tear film. Schirmer test results demonstrated lower tear production correlated with diabetes duration and poor glycemic control. Tear meniscus height was modestly reduced in T2DM, reflecting decreased tear reservoir volume. Subjective symptoms, as measured by OSDI, were generally higher among patients with T2DM, suggesting greater ocular surface discomfort. **Conclusions**: T2DM is strongly associated with tear film instability, reduced tear secretion, and increased dry eye symptoms. These findings suggest that diabetic care should include routine ocular surface assessment and highlight the need for standardized, longitudinal investigations.

## 1. Introduction

Diabetes mellitus (DM) is a major public health concern, currently affecting over 240 million people worldwide, with projections indicating a rise to nearly 370 million cases by 2030 [1]. As the prevalence of this chronic metabolic disorder continues to grow, so does the burden of its complications on patients and healthcare systems [2,3,4]. While diabetic retinopathy has long been recognized as the most vision-threatening ocular complication of DM, there is increasing evidence that the disease also impacts the ocular surface and tear film, often leading to symptoms of dry eye disease and visual discomfort [5,6].

The tear film plays a pivotal role in maintaining ocular surface integrity, visual quality, and patient comfort. Its stability is ensured through a dynamic balance of tear secretion, distribution, and clearance, involving the lacrimal glands, meibomian glands, corneal and conjunctival epithelia, and associated neural regulation. Growing evidence indicates that sustained hyperglycemia impairs meibomian gland activity by disrupting lipid synthesis and secretion, leading to qualitative and compositional changes in the tear film’s lipid layer [7,8]. These metabolic disturbances contribute to tear film instability and increased evaporation, which predispose patients to ocular surface disorders such as dry eye disease (DED), meibomian gland dysfunction, and blepharitis [9,10,11,12]. Clinically, these conditions manifest as irritation, burning, dryness, and fluctuating vision [13,14]. On a pathophysiological level, chronic alterations in tear film homeostasis under hyperglycemic stress can trigger oxidative damage, microvascular dysfunction, and low-grade inflammation of the ocular surface. Over time, this cascade may compromise the integrity of the cornea and conjunctiva, thereby increasing the risk of progressive and irreversible visual impairment [15,16,17].

Clinical investigations have reported variable results regarding the extent and nature of tear film abnormalities in patients with DM. To assess these changes, standard diagnostic tools have been widely applied, such as the Schirmer test for tear secretion [18], non-invasive tear break-up time (NITBUT) for tear stability [19,20], tear meniscus height (TMH) and volume for tear reservoir [21], and the Ocular Surface Disease Index (OSDI) questionnaire for subjective symptoms [22]. However, findings across studies remain inconsistent, with some reporting significant deterioration in tear film parameters among individuals with DM, whereas others observe only subtle or no differences compared to healthy controls [6,23].

Recent technological advances in corneal topography and ocular surface imaging have also enabled more precise assessment of tear film parameters. Modern topographers provide automated, non-invasive measurements of NIBUT and TMH, often offering better repeatability than traditional fluorescein-based TBUT [24]. Studies show that NITBUT correlates strongly with clinical dry eye severity and is typically shorter in patients with tear film instability. In addition, it allows detailed visualization of tear film break-up patterns, enabling characterization of early versus progressive break-up zones, which may reflect different underlying deficiencies [24,25]. Recent experiments comparing different procedures have demonstrated that non-invasive methods generally show longer and more consistent break-up times, supporting the growing use of objective topography-based assessments in tear film evaluation [26]. TMH assessment using OCT or keratography has also emerged as a reliable marker of tear volume [27]. Interferometric techniques also complement these assessments by quantifying lipid layer thickness, demonstrating that thinner lipid layers correlate with increased evaporation and tear film instability [28,29]. Moreover, a recent literature review of innovative ocular surface diagnostic devices concluded that, although these systems can simultaneously assess osmolarity, NITBUT, TMH, lipid layer and meibomian gland status, their diagnostic performance remains heterogeneous and they should complement rather than replace traditional dry eye tests [30]. Together, these newer imaging modalities provide a more comprehensive evaluation of tear film function and help explain the variability observed across traditional subjective and invasive tear tests.

Given the growing prevalence of DM worldwide and the impact of ocular surface alterations on the quality of life and visual function [31,32,33], it is important to synthesize current evidence on tear film changes associated with DM. A comprehensive understanding of these alterations may facilitate early diagnosis of ocular surface disease in diabetic patients, improve monitoring of disease progression, and guide targeted interventions.

This systematic review and meta-analysis aims to critically evaluate and summarize the available literature on tear film parameters in patients with type 2 diabetes mellitus (T2DM). By combining qualitative synthesis with quantitative analysis, this study aims to clarify the relationship between T2DM and tear film dysfunction and highlight potential avenues for future research and clinical practice.

## 2. Materials and Methods

### 2.1. Protocol and Registration

This systematic review and meta-analysis was conducted in accordance with the Preferred Reporting Items for Systematic Reviews and Meta-Analyses (PRISMA) 2020 guidelines [34]. The protocol was registered in the PROSPERO database (ID 1152361).

### 2.2. Eligibility Criteria

In this review, we included observational studies (cross-sectional, case-control, or cohort studies) that compared tear film parameters between adult patients with T2DM and non-diabetic controls. Studies were considered eligible if they were original studies published in English between 30 June 2016 and 30 June 2025 that reported at least one of the following outcomes: NITBUT, invasive tear breakup time (ITBUT), TMH, OSDI, Schirmer test 1 or 2. The exclusion criteria were the following: studies lacking a healthy control group, studies involving contact lens wearers, individuals with other ocular surface diseases, or participants using topical/systemic medications known to influence tear film physiology, studies with insufficient quantitative data for meta-analysis, case reports, conference abstracts, animal studies, in vitro studies, preprints, reviews, and editorials. Studies involving T1DM were also excluded as T1DM differs fundamentally from T2DM in etiology, age of onset, and autoimmune mechanisms, which may independently affect tear film physiology. Restricting the review to T2DM ensures a more clinically homogeneous population and clearer interpretation of type 2 diabetes-related tear film changes.

### 2.3. Search Strategy

A comprehensive literature search was performed in PubMed, Google Scholar, Scopus and Web of Science. The search relied on keywords and Medical Subject Headings (MeSH) terms such as diabetes mellitus, type 2 diabetes and tear film, dry eye, NITBUT, ITBUT, TMH, OSDI or Schirmer test. The detailed Boolean expressions of our search terms are provided in the Supplementary Materials, Table S1. After removing duplicates, two independent reviewers screened titles and abstracts for relevance. Disagreements were resolved by retrieving the full texts of potentially eligible studies and consultation with a third reviewer.

### 2.4. Data Extraction

Data were extracted independently by two reviewers using a standardized form. For each included study, data were extracted for the following predefined outcomes: NITBUT, ITBUT, Schirmer’s test, OSDI, and TMH. Additional variables included the list of authors, year of publication, country, study design, sample size, age, sex and duration of diabetes, mean HbA1c.

### 2.5. Methodological Variability

Differences in tear film assessment techniques among the included studies may influence the interpretation of tear film physiology in T2DM. NITBUT typically provides longer and more stable values compared with ITBUT, as fluorescein itself can disrupt tear film stability and accelerate breakup. TMH measurements also vary depending on the device used, as each device captures slightly different anatomical boundaries. These methodological differences do not change the overall direction of associations between DM and tear dysfunction; however, they contribute to inter-study heterogeneity and must be considered when interpreting patterns of tear film pathophysiology across studies.

### 2.6. Risk of Bias Assessment

The quality of the included studies was assessed using the Newcastle–Ottawa Scale (NOS) for observational studies, evaluating selection, comparability, and outcome domains. Scores of 0–3, 4–6, and 7–9 were considered low, moderate, and high quality, respectively.

### 2.7. Statistical Analysis

All quantitative outcomes were reported as mean ± standard deviation (SD). For studies that reported results stratified by subgroups, we pooled the subgroup means and SDs to derive an overall mean and SD for the diabetic group, using standard formulas for combining summary statistics. When outcomes were reported as median and interquartile range, values were transformed into approximate means and SDs according to validated methods, using the validated methods of Wan et al. [35] and Luo et al. [36]. These formulas allow estimation of mean ± SD from median, IQR, and sample size, ensuring comparability across studies.

Meta-analyses were performed using standardized mean differences (SMDs) with 95% confidence intervals (CIs) for continuous outcomes to account for variability in measurement scales across studies. A random-effects model was chosen a priori, given the expected clinical and methodological diversity.

Between-study heterogeneity was assessed using the χ^2^ test (with *p* < 0.10 indicating significance) and quantified using the I^2^ statistic, with thresholds of 25%, 50%, and 75% interpreted as low, moderate, and high heterogeneity, respectively.

Publication bias was evaluated through visual inspection of funnel plots and formally tested using Egger’s regression asymmetry test. All statistical analyses were conducted using Review Manager version 5.4 (Cocharne RevMan^®^, London, UK). A two-tailed *p*-value < 0.05 was considered statistically significant.

## 3. Results

**Figure 1:**
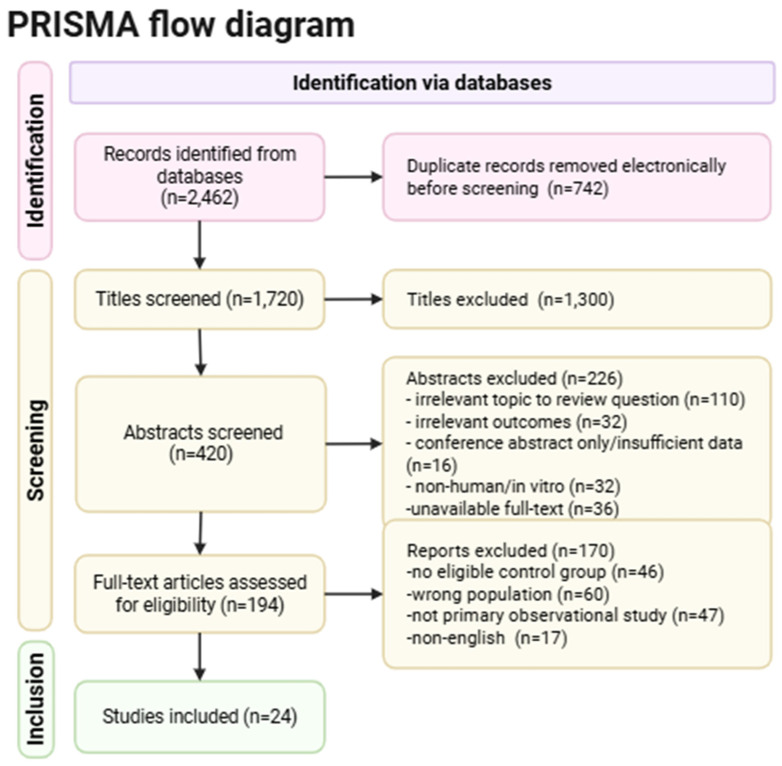
PRISMA 2020 flow diagram illustrating the structured literature search process.

Shown in Figure 1 is the PRISMA 2020 flow diagram of literature search and handling.

Twenty-four studies were included in this systematic review and meta-analysis (Table 1). Overall, they reported data from 3500 eyes of participants with and without T2DM. The majority of the included studies originated from China (15 studies, 2412 eyes), followed by India (3 studies, 572 eyes), Turkey (2 studies, 200 eyes), the United States (1 study, 40 eyes), Colombia (1 study, 73 eyes), Hungary (1 study, 83 eyes), Brazil (1 study, 60 eyes), and Ukraine (1 study, 60 eyes).

The ages of study participants ranged from 25.6 ± 14.6 to 68.96 ± 8.46 years. The duration of T2DM was reported in 13 studies, ranging from 5.0 ± 5.5 to 19.0 ± 10.1 years. Glycemic control, assessed through HbA1c, was reported in 13 studies and ranged from 5.5 ± 0.3% in controls to 9.6 ± 2.7% in patients with T2DM. Most studies followed a cross-sectional or case-control design. Control groups typically consisted of age-matched individuals without diabetes or ocular surface disease.

### 3.1. Invasive Tear Break-Up Time

A total of 14 studies investigated invasive tear break-up time (ITBUT) in patients with T2DM compared to non-diabetic controls (Table 2). A total of 1048 subjects in the experimental cohort and 746 subjects in the control cohort were analyzed. Most reports demonstrated significantly shorter ITBUT values in the diabetic groups, suggesting impaired tear film stability, although a minority of studies found no significant differences.

Fan et al. [51] found that T2DM patients with poor glycemic control (HbA1c ≥ 7%) had significantly shorter ITBUT than both well-controlled diabetic and non-diabetic participants, with differences reaching statistical significance against both comparator groups. Similarly, Kesarwani et al. [39] reported mean ITBUT values of 9.65 ± 2.87 s in diabetics versus 14.54 ± 2.92 s in controls, while Lin et al. [40] observed shorter times in diabetes patients (3.79 ± 2.25 s) compared to non-diabetics (3.99 ± 2.60 s), although the latter difference was not statistically significant. Manchikanti et al. [54] likewise noted a striking reduction in ITBUT due to T2DM. In line with these observations, Mangoli et al. [59] reported lower mean ITBUT in diabetics (12.43 ± 5.32 s) than in control subjects (16.46 ± 4.55 s), with a higher proportion of diabetics showing marginal or low tear film stability. Zou et al. [50] confirmed this pattern, with patients having diabetes and dry eye showing severely reduced ITBUT (3.58 ± 0.81 s) compared to both diabetics without dry eye (10.97 ± 2.00 s) and healthy controls (11.63 ± 1.78 s, *p* < 0.001). Wu et al. [57] also documented significant impairment (6.25 ± 1.99 s in diabetic subjects vs. 10.87 ± 1.79 s in controls, *p* < 0.001).

Conversely, some studies reported no significant difference or even paradoxical findings. Stuard et al. [42] found no significant difference in ITBUT between their study groups, noting that ITBUT values were slightly lower in controls (median 4.8 s) compared to diabetics (5.5 s), though both groups were within the borderline-to-abnormal range. Tóth et al. [55] presented a more complex picture, reporting ITBUT values that varied according to subgroup: while some participants with T2DM exhibited lower tear stability than obese controls, others had comparable or even higher times, with substantial variability across the sample.

**Figure 2:**
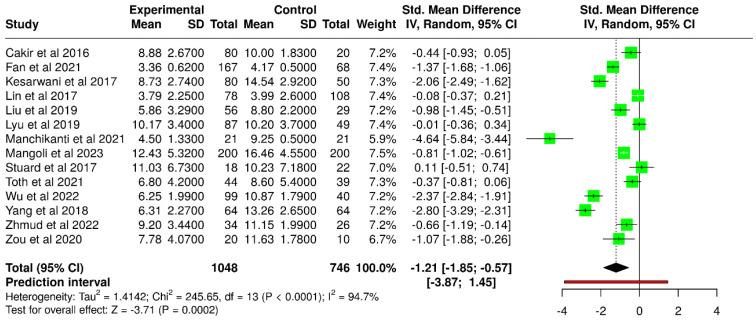
Forest plot of included studies assessing ITBUT test results in diabetic versus non-diabetic participants [37,39,40,42,43,45,46,50,51,54,55,57,58,59].

Based on the random-effects model using the inverse variance method to compare SMD, a statistically significant difference was observed between the diabetic and control groups (Figure 2). The pooled SMD was −1.21 (95% CI: −1.85 to −0.57), with the overall effect reaching statistical significance (*p* < 0.05). However, a high level of heterogeneity was detected across studies (*p* < 0.01), with an I^2^ value of 95%, indicating that most of the variability in results reflects true differences in study outcomes rather than random error.

**Figure 3:**
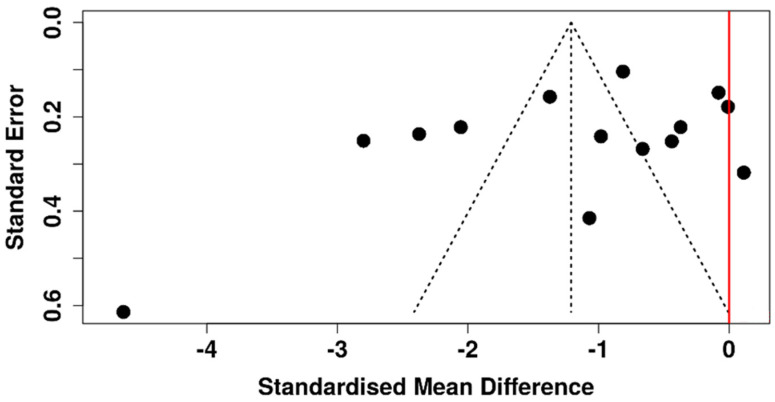
Funnel plot assessing potential publication bias among studies evaluating ITBUT in diabetic versus non-diabetic participants. The red line represents the line of no effect, whereas the dashed vertical line represents the pooled effect estimate.

The corresponding funnel plot shows a symmetrical distribution of studies, suggesting no evidence of publication bias (Figure 3). This observation is further supported by Egger’s test, which did not demonstrate significant asymmetry (intercept: −3.85, 95% CI: −9.64 to 1.93, t = −1.306, *p* = 0.216).

### 3.2. Non-Invasive Tear Break-Up Time

Ten studies evaluated NITBUT in diabetic and non-diabetic populations, with a total of 863 subjects in the experimental groups and 669 subjects in the control groups (Table 3).

Han et al. [52] reported a progressive decline in average NITBUT with increasing severity of diabetic retinopathy, from 10.17 ± 3.91 s in controls to 9.82 ± 3.64 s in non-diabetic retinopathy, 6.10 ± 3.11 s in non-proliferative DR, and 5.96 ± 3.31 s in proliferative DR, with all comparisons significant (*p* < 0.01). Yang et al. [60] similarly documented severe impairment, with NITBUT averaging 4.15 ± 0.98 s in diabetic patients with dry eye, compared to 14.34 ± 5.97 s in diabetics without dry eye and 16.80 ± 4.74 s in healthy controls. Yu et al. [38] reported shorter NITBUT in diabetic subjects (4.44 ± 2.40 s) versus controls (8.42 ± 3.79 s, *p* < 0.001). Trindade et al. [56] found mean NITBUT of 9.34 ± 6.82 s in diabetics, lower than in non-diabetic controls (13.39 ± 7.00 s), with particularly short times in those with combined arthropathy and diabetes (7.59 ± 4.48 s). Sandra et al. [47] also observed slightly reduced NITBUT values in diabetes (2.47 ± 1.3 s) versus control (2.9 ± 1.2 s), although this difference was not significant.

By contrast, other studies reported little or no difference. Liang et al. [53] found mean NITBUT values of 9.48 ± 3.79 s in diabetics compared to 9.09 ± 3.91 s in controls (*p* = 0.462). Lin et al. [40] reported similar averages (8.59 ± 4.94 s in diabetes vs. 9.53 ± 5.61 s in non-diabetics, *p* = 0.44). Yusufu et al. [44] observed no significant baseline difference (13.1 ± 4.7 s in diabetics vs. 12.2 ± 6.4 s in controls, *p* = 0.433). Zeng et al. [48] likewise reported no group differences, with NITBUT values broadly overlapping across diabetic and non-diabetic participants (≈9–10 s).

**Figure 4:**
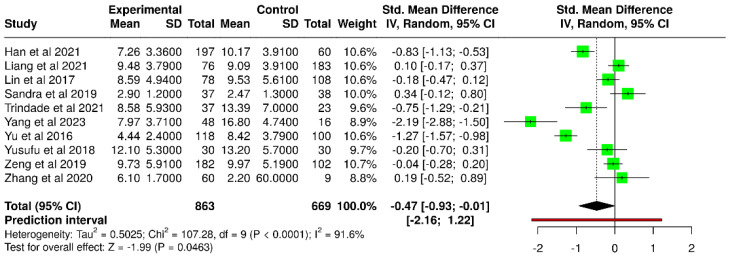
Forest plot of included studies assessing NITBUT test results in diabetic versus non-diabetic participants [38,40,44,47,48,49,52,53,56,60].

Based on the random-effects model with the inverse variance method, a statistically significant difference was identified between diabetic and non-diabetic groups. The pooled SMD was −0.47 (95% CI: −0.93 to −0.01), with the overall effect reaching statistical significance (*p* < 0.05) (Figure 4). However, substantial heterogeneity was present (*p* < 0.01), with an I^2^ value of 92%.

**Figure 5:**
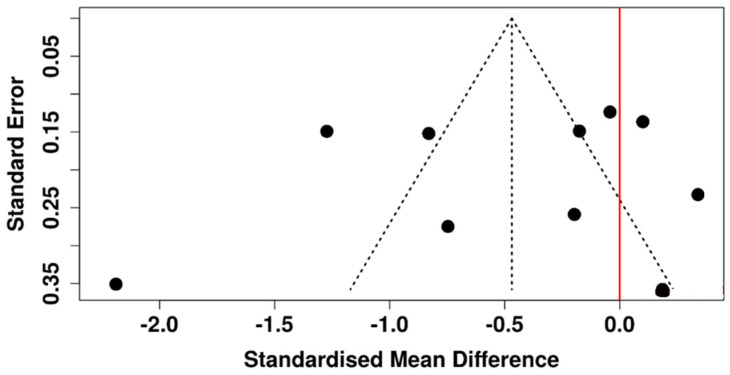
Funnel plot assessing potential publication bias among studies evaluating NITBUT test results in diabetic versus non-diabetic participants.

The funnel plot appeared symmetrical, indicating no evidence of publication bias (Figure 5). This observation was supported by Egger’s test, which did not reveal significant asymmetry (intercept: −1.91, 95% CI: −8.53 to 4.71, t = −0.566, *p* = 0.587).

### 3.3. Schirmer’s Test

Altogether 19 studies were analyzed with a total of 1331 subjects in the T2DM groups and 988 subjects in the control groups and marked reductions in tear secretion were consistently observed in several studies (Table 4).

Fan et al. [51] reported median Schirmer values of 5.0 mm in T2DM patients with poor glycemic control (HbA1c ≥ 7%) and 4.5 mm in well-controlled diabetics, compared to 10.0 mm in non-diabetics. The differences were significant against the control group (*p* = 0.001), but not between diabetic subgroups. Kesarwani et al. [39] similarly found lower tear production in both DR (9.54 ± 5.32 mm) and non-diabetic retinopathy (9.95 ± 4.56 mm) compared to markedly higher values in controls (25.84 ± 7.32 mm). Manchikanti et al. [54] also observed reduced secretion (9.57 ± 9.33 mm in diabetics vs. 22.57 ± 6.79 mm in controls, *p* < 0.01). Zou et al. [50] reported profound tear secretion impairment in diabetics with dry eye (3.30 ± 1.57 mm) relative to diabetics without dry eye (14.80 ± 6.63 mm) and healthy controls (18.90 ± 7.82 mm, *p* < 0.001). Wu et al. [57] confirmed this trend, with diabetics displaying significantly lower Schirmer values (9.59 ± 3.17 mm) than controls (13.58 ± 2.92 mm, *p* < 0.001). Qu et al. [41] also documented reduced secretion in both diabetic groups (≈6.7–6.8 mm) relative to controls (13.8 mm, *p* < 0.01).

Evidence of severity- or duration-related decline in tear secretion was also reported. Lyu et al. [46] demonstrated that T2DM patients with longer disease duration (>10 years) had significantly lower Schirmer values than both controls and those with shorter disease duration. Yang et al. [43] found reduced secretion in diabetic patients with concomitant dry eye (6.65 ± 3.46 mm) compared to diabetic patients without dry eye symptoms (9.78 ± 2.90 mm) and healthy controls (14.0 ± 5.98 mm). The study of Zeng et al. [48] found progressively lower Schirmer values with increasing diabetes duration.

By contrast, several studies reported no significant differences. Stuard et al. [42] observed similar values between diabetics (19.1 ± 8.2 mm) and controls (17.8 ± 7.9 mm, *p* = 0.510), in line with Liang et al. [53] (8.17 ± 6.72 mm vs. 9.26 ± 5.56 mm, *p* = 0.180), Lin et al. [40] (5.57 ± 4.70 mm vs. 6.55 ± 5.93 mm, *p* = 0.431), and Yusufu et al. [44] (12.3 ± 6.6 mm vs. 12.7 ± 6.0 mm, *p* = 0.865).

**Figure 6:**
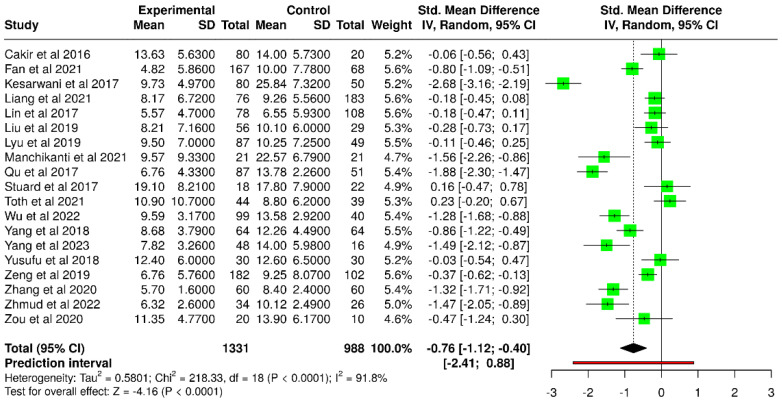
Forest plot of included studies assessing Schirmer’s test in diabetic versus non-diabetic participants [37,39,40,41,42,43,44,45,46,48,49,50,51,53,54,55,57,58,60].

In the framework of the random-effects model, a statistically significant difference was observed between the diabetic and control groups. The pooled SMD was −0.76 (95% CI: −1.12 to −0.40), and the test for overall effect reached statistical significance (*p* < 0.05) (Figure 6). Nevertheless, considerable heterogeneity was detected (*p* < 0.01), with an I^2^ value of 92%, indicating that most of the variability among studies reflected true differences in effect size and/or direction as opposed to random variation.

**Figure 7:**
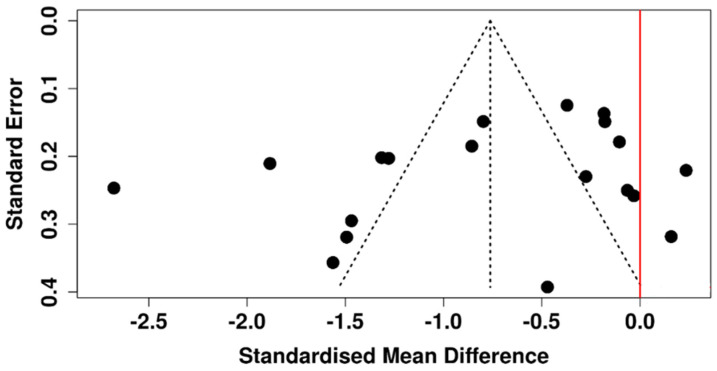
Funnel plot evaluating potential publication bias among studies assessing Schirmer test in diabetic versus non-diabetic participants.

The funnel plot shown in Figure 7 symmetrical, indicating no evidence of publication bias. Egger’s test further confirmed the absence of significant asymmetry (intercept: −3.43, 95% CI: −8.46 to 1.59, t = −1.34, *p* = 0.198).

### 3.4. Ocular Surface Disease Index

Fifteen studies investigated OSDI in diabetic versus non-diabetic populations, with a total of 996 subjects in experimental groups and 807 subjects in control groups (Table 5). Several studies demonstrated a marked deterioration of OSDI scores in T2DM. Manchikanti et al. [54] reported a mean OSDI of 42.95 ± 17.38 in diabetic subjects compared to 16.75 ± 5.45 in control participants (*p* < 0.01). Wu et al. [57] found higher scores in the diabetic group (19.98 ± 8.91) relative to the control group (10.31 ± 1.45) and confirmed further increases in a dry eye sample (24.94 ± 5.22, *p* < 0.001). Yu et al. [38] similarly documented higher OSDI in diabetes (23.02 ± 13.13) versus controls (12.11 ± 6.48, *p* < 0.001). Sandra et al. [47] observed a significantly greater mean OSDI in T2DM (22.2 ± 7.93) compared to control subjects (16.2 ± 10.60, *p* < 0.05), with a higher proportion of diabetic patients meeting the threshold for dry eye diagnosis (78% vs. 47%). Han et al. [52] demonstrated progressive deterioration of the OSDI score across the spectrum of diabetic retinopathy: it rose from 4.00 ± 1.44 in controls to 9.96 ± 2.14 in non-diabetic retinopathy patients, 16.15 ± 2.25 in non-proliferative DR, and 32.81 ± 2.87 in proliferative DR (*p* < 0.01). Trindade et al. [56] also reported elevated OSDI (≈20–25 in diabetics vs. ≈9 in controls, *p* = 0.037). Zhang et al. [49] and Qu et al. [41] both found significant increases in diabetic patients, with Qu et al. showing particularly pronounced differences (≈30 in diabetic groups vs. ≈4 in controls).

In contrast, other investigators reported milder or non-significant changes in OSDI associated with T2DM. Stuard et al. [42] found median OSDI slightly higher in diabetics (10.4) versus controls (3.2), but not statistically significant (*p* = 0.256). Likewise, Liang et al. [53] (21.9 ± 12.7 vs. 20.2 ± 11.1, *p* = 0.286) and Lin et al. [40] (12.8 ± 13.9 vs. 13.6 ± 16.4, *p* = 0.975) reported no difference. Yusufu et al. [44] also did not detect consistent differences across time points in their intervention (9.2 ± 11.0 vs. 8.9 ± 12.9).

**Figure 8:**
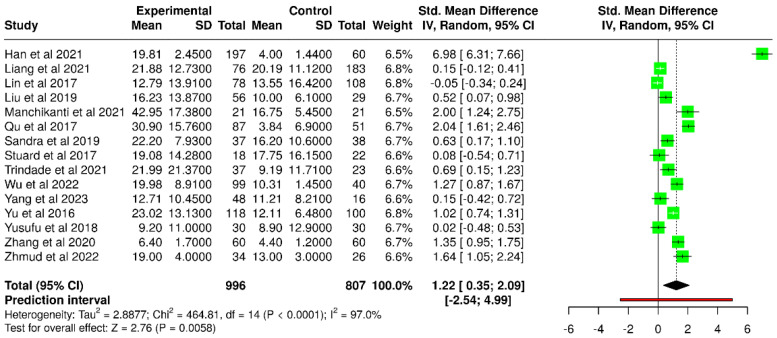
Forest plot of included studies evaluating OSDI scores in T2DM patients versus control participants [38,40,41,42,44,45,47,49,52,53,54,56,57,58,60].

Within the random-effects model, a statistically significant difference was detected between the diabetic and control groups. The pooled SMD was 1.22 (95% CI: 0.35 to 2.09), with the overall effect reaching statistical significance (*p* < 0.05) (Figure 8). There was significant heterogeneity (*p* < 0.01), and the I^2^ value of 97% showed that most variability among trials represented real differences in impact size and/or direction, rather than random error.

**Figure 9:**
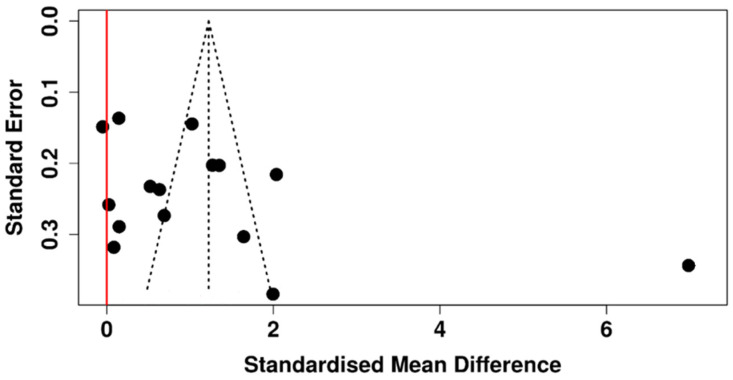
Funnel plot assessing potential publication bias among studies that evaluated OSDI scores in T2DM patients versus non-diabetic subjects.

The associated funnel plot is symmetrical (Figure 9), suggesting a lack of publication bias. Furthermore, Egger’s test did not indicate significant asymmetry (intercept: 8.12, 95% CI: −0.73 to 16.98, t = 1.798, *p* = 0.095).

### 3.5. Tear Meniscus Height

Twelve studies evaluated TMH in patients with T2DM compared with non-diabetic control subjects, with a total of 1086 participants in the diabetic groups and 765 subjects in the control groups (Table 6).

Fan et al. [51] reported that TMH was lowest in patients with poor glycemic control, intermediate in well-controlled diabetes, and highest in non-diabetic controls, with significant differences between diabetics and controls (*p* < 0.001). Han et al. [52] observed a progressive decline in TMH across DR severity stages, from 0.28 ± 0.12 mm in controls to 0.25 ± 0.07 mm in non-diabetic retinopathy, 0.24 ± 0.10 mm in non-proliferative diabetic retinopathy (NPDR), and 0.18 ± 0.55 mm in proliferative diabetic retinopathy (PDR) (*p* < 0.01). Zhang et al. [49] likewise reported lower values in diabetics (≈176 μm) compared to controls (≈191 μm, *p* < 0.01). Yang et al. [60] confirmed reduced TMH in diabetics with dry eye (0.22 ± 0.06 mm) compared to diabetics without dry eye (0.28 ± 0.08 mm) and healthy controls (0.27 ± 0.07 mm).

By contrast, Sandra et al. [47] found no significant difference in TMH between diabetic (207 ± 83 μm) and non-diabetic groups (212 ± 71 μm, *p* = 0.86). Trindade et al. [56] reported a non-significant reduction (0.24 ± 0.08 mm in diabetics vs. 0.30 ± 0.08 mm in controls, *p* = 0.056). Liang et al. [53] (0.13 ± 0.03 mm in both groups, *p* = 0.866), Lin et al. [40] (0.19 ± 0.10 vs. 0.22 ± 0.14 mm, *p* = 0.131), and Zeng et al. [48] (values ≈ 0.22–0.24 mm across groups) also found no differences.

**Figure 10:**
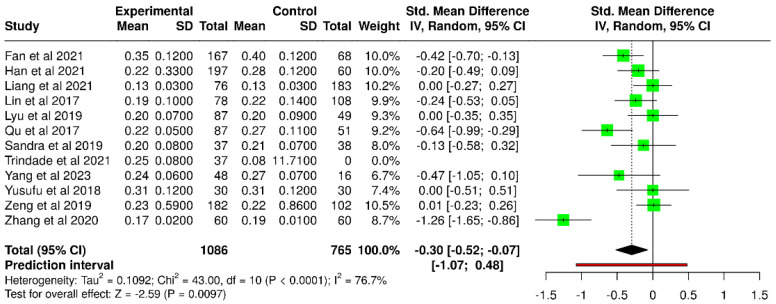
Forest plot evaluating the TMH in diabetic vs. non-diabetic participants [40,41,44,46,47,48,49,51,52,53,56,60].

The random-effects model revealed a statistically significant difference between diabetic and non-diabetic subjects. The pooled SMD was −0.30 (95% CI: −0.52 to −0.07), and the test for the overall effect was statistically significant (Figure 10). With an I^2^ value of 77%, however, there was significant heterogeneity (*p* < 0.01), indicating that the variation among studies was not due to chance but rather represented actual variations in impact size and direction.

**Figure 11:**
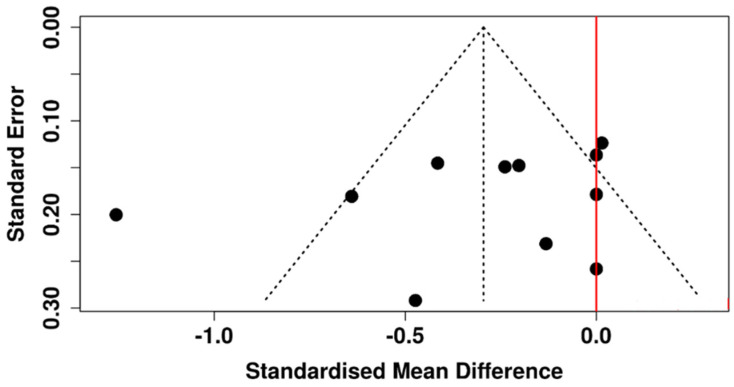
Funnel plot scrutinizing potential publication bias among studies that measured the TMH in participants with T2DM and non-diabetic subjects.

The symmetrical funnel plot of Figure 11 suggests that publication bias is absent, and this conclusion is further supported by Egger’s test (intercept: −2.78, 95% CI: −7.81 to 2.24, t = −1.086, *p* = 0.306).

### 3.6. Risk of Bias Assessment

For each of the included studies, we assessed the risk of bias using a variety of statistical tools, depending on the study design.

Most studies were cross-sectional (*n* = 20); these were assessed using the Newcastle–Ottawa Scale adapted for cross-sectional design. All of them were found to be at low, low/moderate or moderate risk of bias (Table 7).

One randomized controlled trial, Yusufu et al. [44], was evaluated using the RoB 2 tool and was judged to have a low-to-moderate risk of bias. One non-randomized interventional study [26] was assessed using the ROBINS-I tool and was deemed to have an overall low-to-moderate risk of bias. While confounding, selection, and outcome measurement were rated as moderate, other domains were low risk, suggesting acceptable methodological quality with some limitations. One case–control study [42] was assessed with the Newcastle–Ottawa Scale adapted for case–control design. It showed a moderate overall risk of bias, with outcome ascertainment at low risk but raised concerns regarding control selection and comparability.

## 4. Discussion

The present systematic review and meta-analysis synthesized evidence from 24 observational studies assessing tear film parameters in patients with T2DM compared with age-matched, non-diabetic control subjects. Our findings demonstrate that T2DM is consistently associated with abnormalities across multiple tear film characteristics, such as tear film stability, tear secretion, and tear reservoir parameters, as well as subjective symptoms related to dry eye disease.

The origins of dry eye symptoms in diabetes are not yet fully understood. One proposed mechanism for the emergence of the dry eye condition is microvascular damage within the lacrimal gland, which may compromise its ability to produce tears [61]. In addition, it has been established that autonomic neuropathy associated with T2DM can further impair glandular regulation and tear secretion [62]. Corneal sensory neuropathy, another frequent complication of T2DM, may reduce corneal sensitivity and disrupt the reflex arc that stimulates tear production [63,64]. Chronic hyperglycemia promotes oxidative stress and accumulation of advanced glycation end products, which activate inflammatory cascades on the ocular surface and alter the composition of both the aqueous and mucin layers [65]. Meibomian gland dysfunction, more common in diabetics due to ductal epithelial damage, lipid compositional changes, and low-grade inflammation. compromises the lipid layer, increasing evaporative loss and contributing to shortened NITBUT and ITBUT [66]. Reduced blink quality, impaired lid apposition, and subtle eyelid structural changes seen in diabetes further destabilize the tear film by disrupting its even distribution [40]. Taken together, these metabolic, inflammatory, vascular and neural changes can bring about both quantitative and qualitative tear film abnormalities, ultimately leading to ocular surface instability and dry eye symptoms.

Tear film stability, characterized by the ITBUT and NITBUT, was significantly reduced in diabetic populations, with several studies reporting values well below those of non-diabetic controls. In the meta-analysis of Kuo et al. [6], both ITBUT and NITBUT were consistently reduced in individuals with diabetes compared to non-diabetic controls. ITBUT was significantly shorter across 41 studies, with subgroup analyses confirming this reduction in both type 1 and type 2 DM, as well as in unclassified diabetes, though no significant difference was observed in gestational diabetes. NITBUT findings similarly demonstrated progressive decline with increasing severity of diabetic retinopathy and poorer glycemic control. Another analysis demonstrated that ITBUT was significantly reduced in patients with diabetes mellitus compared to non-diabetic controls, with a pooled weighted mean difference of −4.44 s (*p* < 10^−5^). Subgroup analyses revealed a progressive decline in ITBUT with advancing DR, as values were lower in NPDR than in non-diabetic retinopathy, and lowest in PDR [67].

Tear secretion, assessed by the Schirmer test, was also found to be impaired in diabetes. Many studies reported markedly lower Schirmer test results among diabetic patients, with some suggesting a relationship between reduced secretion and longer disease duration or poor glycemic control. Nonetheless, a subset of studies failed to identify significant differences. In the study of Kuo et al. [6], patients with diabetes demonstrated significantly lower tear secretion values compared with non-diabetic controls (SMD: −0.45, 95% CI: −0.64 to −0.26, I^2^ = 90%). Subgroup analyses revealed that this reduction was present in both type 1 and type 2 diabetes, whereas no significant differences were observed in unclassified diabetes or gestational diabetes. Importantly, when stratified by glycemic control, only patients with poor HbA1c control demonstrated significantly reduced Schirmer test scores, whereas those with well-controlled HbA1c had tear secretion levels comparable to controls [6].

When comparing tear film characteristics and tear secretion across regions, noteworthy differences can be observed between Asian and European study samples. Most Asian studies consistently reported significantly shorter ITBUT/NITBUT values and reduced Schirmer test scores in patients with diabetes versus non-diabetic subjects. In contrast, European studies provided mixed results. Asian study groups were also found to display a higher prevalence of dry eye disease [68,69]. These geographic discrepancies may be explained by several factors, including differences between population-level characteristics (such as race, ethnicity, and lifestyle), environmental factors, and healthcare systems.

Subjective symptoms of ocular surface disease, measured in terms of OSDI scores, were found to be more severe among diabetic participants. Several studies have shown that, in diabetic patients, significantly higher OSDI scores are associated with elevated HbA1c levels [17]. Higher HbA1c levels were strongly associated with the presence and severity of dry eye symptoms, and OSDI scores increased in parallel with disease severity (7.9 in non-dry eye vs. 57 in severe dry eye, *p* < 0.0001) [11].

Symptom–sign discordance is a well-recognized feature of diabetes-related ocular surface disease. Many patients with T2DM demonstrate substantial tear film instability, reduced tear secretion, or altered tear film composition, yet report only mild or inconsistent symptoms. T2DM impairs trigeminal and autonomic neural feedback pathways, resulting in blunted reflex tearing and delayed detection of ocular surface stress [70]. Additional mechanisms may further explain why tear film abnormalities in T2DM do not always parallel symptom severity. Evidence shows that longer diabetes duration is associated with progressive loss of corneal nerve structure and function, including reduced corneal sensitivity, reduced corneal nerve density, shorter corneal nerve fiber length, and diminished inferior whorl length [46,71]. These neural changes have been shown to correlate strongly with reduced reflex tearing and lower Schirmer scores, particularly in individuals with diabetes lasting more than 10 years [46]. Importantly, patients with long-standing diabetes may experience fewer or no symptoms despite significant tear film deficiency, likely because decreased corneal sensitivity blunts the perception of ocular surface irritation [46]. Findings from another study also support a clear symptom–sign mismatch in diabetes. Patients with diabetes reported significantly fewer dry eye and ocular pain symptoms than controls, despite showing more anatomical abnormalities. Importantly, tear parameters and corneal staining did not differ across groups, suggesting that reduced symptom reporting in diabetes may reflect altered sensory function rather than milder disease [16].

TMH findings were less consistent in our systematic review. While some reports documented significant reductions in diabetic groups, others observed no differences. The study of Meng et al. [72] demonstrated that patients with elevated glucose levels (≥7 mmol/L) had significantly smaller TMH (0.15 ± 0.04 mm vs. 0.22 ± 0.07 mm, *p* < 0.001), and TMH was negatively correlated with blood glucose level (*p* < 0.001). In one study of 88 patients with diabetes, the TMH was significantly reduced compared with non-diabetic controls (0.6 vs. 0.8) [73].

Han et al. [52] showed that patients with proliferative DR demonstrated shorter NITBUT values, reduced TMH, and higher OSDI scores compared with those with non-proliferative DR. This pattern is biologically plausible as increasing DR severity is associated with greater microvascular ischemia, autonomic neuropathy, and sub-basal corneal nerve fiber loss [74,75,76], all of which impair lacrimal gland function, reduce blink efficiency, and destabilize the tear film [77,78]. Advanced DR is also linked to chronic low-grade inflammation and higher tear cytokine levels, which may accelerate tear evaporation and epithelial damage. [79,80]. Beyond the retinopathy stage alone, glycemic control and diabetes duration also appear to influence ocular surface impairment [81]. Poorly controlled hyperglycemia exacerbates oxidative stress and microvascular dysfunction within the lacrimal glands, accelerating reductions in aqueous tear secretion [82], while long-standing diabetes increases cumulative neural damage affecting both autonomic innervation and corneal sensory fibers [83]. However, studies have been inconsistent about how HbA1C might influence the presence of dry eye disease symptoms, with some of them reporting no significant correlation [84].

This systematic review and meta-analysis has several strong points. By synthesizing data from 24 studies conducted across diverse populations, it provides a comprehensive overview of the impact of type 2 diabetes mellitus on tear film characteristics. Importantly, a broad range of tear film parameters, encompassing NITBUT, ITBUT Schirmer test, TMH, and OSDI scores, were evaluated, allowing for an integrated assessment of both objective and subjective aspects of ocular surface dysfunction.

Nevertheless, this article also has limitations. First, most of the included studies were cross-sectional in design, which precludes causal inferences and limits the ability to evaluate longitudinal changes in tear film parameters with T2DM progression. Second, although we conducted subgroup analyses, the heterogeneity remained substantial across studies, most likely due to variations in study design, population characteristics, and the techniques used to measure tear film parameters. Differences in how BUT, tear volume, or symptoms were assessed, as well as the specific devices and protocols adopted in each study, can naturally lead to variability in reported outcomes. Environmental conditions and regional practice patterns may have also contributed, given that several studies originated from the same geographical area. These factors highlight the need for future research to adopt more uniform diagnostic approaches and standardized measurement protocols to improve comparability across populations. Third, glycemic control and duration of diabetes were inconsistently reported in the publications reviewed here, making it difficult to assess their roles as modifiers of tear film dysfunction fully.

## 5. Conclusions

This article demonstrates that T2DM is consistently associated with significant alterations in tear film stability, secretion, and subjective visual comfort. Across multiple studies, diabetic participants exhibited shorter tear break-up times, lower Schirmer test values, and modest reductions in tear meniscus height, together with higher OSDI scores indicating greater ocular surface discomfort. These findings suggest that chronic hyperglycemia adversely affects the lacrimal and meibomian glands, contributing to an increased prevalence of dry eye disease in diabetic populations. Routine screening for ocular surface disease should be incorporated into comprehensive diabetic eye care, as early identification and management may prevent visual discomfort and improve quality of life in patients with diabetes.

## Figures and Tables

**Table 1:** Overview of included studies.

Study [Reference]	Country	Study Period	Study Design	Groups	Sample Size	Age	Sex M/F	Duration of DM	Mean HbA1c Level
Year					(No. of Patients (Eyes)	(Years, Mean ± SD)	(No.)	(Years, Mean ± SD)	(%, Mean ± SD)
Cakir et al. [37]	Turkey	N/A	Cross-sectional	T2DM with OAD	20 (40)	53.3 ± 6.8	8/12	7	7.6 ± 0.2
2016				T2DM with insulin	20 (40)	52.3 ± 7.0	6/14	6	7.7 ± 0.2
Yu et al. [38]	China	October 2014–November 2015	Case–control	T2DM	118 (118)	59.7 ± 7.8	58/60	N/A	N/A
2016				CG	100 (100)	60.3 ± 7.6	52/48	-	-
Kesarwani et al. [39] 2017	India	N/A	Case–control	T2DM without DR	29 (38)	53.0 ± 5.6	14/15	N/A	N/A
				T2DM with DR	24 (42)	52.5 ± 4.8	12/12	N/A	N/A
				CG	30 (50)	51.7 ± 4.8	15/15	-	-
Lin et al. [40]	China	May–December 2015	Prospective	T2DM	39 (78)	67.1 ± 1.5	16/23	9.1 ± 5.4	N/A
2017			Case–control	CG	54 (108)	67.2 ± 1.7	23/31	-	-
Qu et al. [41] 2017	China	March 2015–November 2016	Prospective Case–control	T2DM without CFS	48 (48)	60.5 ± 8.4	14/34	13.4 ± 8.3	7.7 ± 1.1
				T2DM with CFS	39 (39)	63.8 ± 10.9	14/25	13.9 ± 5.2	7.8 ± 1.8
				CG	51 (51)	61.5 ± 10.2	18/33	-	-
Stuard et al. [42]	USA	N/A	Case–control	T2DM	18 (18)	58.8 ± 10.2	6/12	N/A	7.7 ± 1.0
2017				CG	22 (22)	53.3 ± 9.7	10/12	-	5.7 ± 0.4
Yang et al. [43]	China	October 2015–June 2017	Case–control	T2DM	32 (64)	53.2 ± 3.8	13/19	N/A	9.6 ± 2.7
2018				CG	32 (64)	54.7 ± 3.4	14/18	-	7.6 ± 2.5
Yusufu et al. [44]	China	December 2014–May 2015	Prospective	T2DM	30 (30)	N/A	N/A	N/A	N/A
2018			Case–control	CG	30 (30)	-	-	-	-
Liu et al. [45] 2019	China	January–June 2018	Case–control	T2DM without DED	24 (24)	63.5 ± 10.1	7/17	12.3 ± 6.8	8.0 ± 1.6
				T2DM with DED	32 (32)	61.8 ± 9.8	14/18	11.3 ± 7.1	7.6 ± 1.5
				CG	29 (29)	62.4 ± 7.5	5/24	-	-
Lyu et al. [46]	China	N/A	Prospective	T2DM	87 (87)	65 ± 6	38/49	14 ± 8	6.9 ± 0.5
2019			Case–control	CG	49 (49)	64 ± 5	17/32	-	-
Sandra et al. [47]	Colombia	N/A	Prospective	T2DM	37 (37)	59 ± 7.7	37/0	7.2 ± 5	6.8 ± 0.7
2019			Case–control	CG	36 (36)	58.5 ± 7.4	36/0	-	-
Zeng et al. [48]	China	July–September 2017	Prospective	T2DM	91 (182)	65.4 ± 6.3	40/51	13.6 ± 8.3	7.0 ± 0.5
2019			Case–control	CG	51 (102)	64.4 ± 5.7	18/33	-	-
Zhang et al. [49]	China	N/A	Case–control	T2DM	60 (60)	63.6 ± 11.0	32/28	N/A	N/A
2020				CG	60 (60)	63.4 ± 10.4	30/30	-	-
Zou et al. [50] 2020 (Adult)	China	August 2017	Cross-sectional	T2DM without DED	10 (10)	57.7 ± 7.2	3/7	5.7 ± 3.0	N/A
				T2DM with DED	10 (10)	58.8 ± 4.3	4/6	12.4 ± 4.5	N/A
				CG	10 (10)	58.0 ± 4.3	3/7	-	-
Fan et al. [51] 2021	China	May–December 2018	Cross-sectional	T2DM with HbA1C < 7%	60 (60)	58.9 ± 10.0	36/24	N/A	N/A
				T2DM with HbA1C > 7%	107 (107)	56.8 ± 10.0	62/45	N/A	N/A
				CG	68 (68)	58.4 ± 13.6	33/35	-	-
Han et al. [52] 2021	China	June 2019–August 2020	Cross-sectional	T2DM without DR	33 (66)	56.5 ± 7.5	16/17	N/A	N/A
				T2DM with NPDR	32 (64)	58.6 ± 9.4	15/17	N/A	N/A
				T2DM with PDR	34 (67)	57.9 ± 8.2	17/17	N/A	N/A
				CG	30 (60)	56.4 ± 9.5	16/14	-	-
Liang et al. [53]	China	December 2019–November 2020	Cross-sectional	T2DM	38 (76)	67.6 ± 9.1	16/22	N/A	N/A
2021				CG	92 (183)	32.8 ± 13.4	31/61	-	-
Manchikanti et al. [54]	India	July 2016–December 2017	Case–control	T2DM	21 (21)	54.6 ± 11.6	19/2	N/A	N/A
2021				CG	21 (21)	51.3 ± 10.7	19/2	-	-
Tóth et al. [55]	Hungary	N/A	Cross-sectional	T2DM	44 (44)	50 ± 7	26/18	N/A	7.3 ± 1.1
2021				CG	39 (39)	53 ± 10	16/23	-	5.5 ± 0.3
Trindade et al. [56]	Brazil	January–September 2019	Cross-sectional	T2DM without CA	21 (21)	60.6 ± 7.9	10/11	16.2 ± 8.7	8.7 ± 1.5
2021				T2DM with CA	16 (16)	57.1 ± 11.8	11/5	19 ± 10.1	8.5 ± 2.2
Wu et al. [57] 2022	China	January–December 2016	Cross sectional	T2DM	99	59.72 ± 6.05	47/52	5.24 ± 3.06	7.37 ± 1.28
				CG with DED	33	59.09 ± 7.25	15/18	-	5.96 ± 0.28
				CG without DED	40	58.55 ± 7.18	21/19	-	5.91 ± 0.19
Zhmud et al. [58]	Ukraine	N/A	Cross-sectional	T2DM	34	68.96 ± 8.46	18/16	6.0 ± 4.8	7.28 ± 0.82
2022				CG	26	63.76 ± 6.67	12/14	-	-
Mangoli et al. [59]	India	N/A	Prospective	T2DM	200	N/A	N/A	N/A	N/A
2023			Cross-sectional	CG	200	-	-	-	-
Yang et al. [60] 2023	China	December 2018–December 2019	Cross-sectional	T2DM with DED	30	64.46 ± 9.29	15/15	12.15 ± 7.45	N/A
				T2DM without DED	18	61.11 ± 6.58	11/7	12.83 ± 6.71	N/A
				CG with DED	26	63.58 ± 8.56	10/16	-	-
				CG without DED	16	60.06 ± 7.18	7/9	-	-

Abbreviations: CG—control group; DED—dry eye disease; DR—diabetic retinopathy; N/A—not available; NPDR—non-proliferative diabetic retinopathy; OAD—oral antidiabetic drugs; PDR—prolierative diabetes mellitus; T2DM—type 2 diabetes mellitus.

**Table 2:** Invasive tear film break-up time (ITBUT) values in diabetic and control groups.

Study	T2DM ITBUT (s)	Number of Subjects	Control ITBUT (s)	Number of Subjects
Çakır et al. [37]	8.88 ± 2.67	80	10 ± 1.83	20
Fan et al. [51]	3.36 ± 0.62	167	4.17 ± 0.50	68
Kesarwani et al. [39]	8.73 ± 2.74	80	14.54 ± 2.92	50
Liu et al. [45]	5.86 ± 3.29	56	8.80 ± 2.20	29
Manchikanti et al. [54]	4.50 ± 1.33	21	9.25 ± 0.50	21
Mangoli et al. [59]	12.43 ± 5.32	200	16.46 ± 4.55	200
Wu et al. [57]	6.25 ± 1.99	99	10.87 ± 1.79	40
Yang et al. [43]	6.31 ± 2.27	64	13.26 ± 2.65	64
Zhmud et al. [58]	9.20 ± 3.44	34	11.15 ± 1.99	26
Zou et al. [50]	7.78 ± 4.07	20	11.63 ± 1.78	10
Lin et al. [40] *	3.79 ± 2.25	78	3.99 ± 2.60	108
Lyu et al. [46] *	10.17 ± 3.40	87	10.20 ± 3.70	49
Stuard et al. [42] *	11.03 ± 6.73	18	10.23 ± 7.18	22
Tóth et al. [55] *	6.80 ± 4.20	44	8.60 ± 5.40	39

Studies marked with an asterisk (*) reported non-significant differences between T2DM and control groups (*p* > 0.05).

**Table 3:** Non-invasive tear film break-up time values in diabetic and control groups.

Study	T2DM NITBUT (s)	Number of Subjects	Control NITBUT (s)	Number of Subjects
Han et al. [52]	7.26 ± 3.36	197	10.17 ± 3.91	60
Trindade et al. [56]	8.58 ± 5.93	37	13.39 ± 7.00	23
Yang et al. [43]	7.97 ± 3.71	48	16.80 ± 4.74	16
Yu et al. [38]	4.44 ± 2.40	118	8.42 ± 3.79	100
Zhang et al. [49]	6.1 ± 1.7	60	9.6 ± 2.2	60
Liang et al. [53] *	9.48 ± 3.79	76	9.09 ± 3.91	183
Lin et al. [40] *	8.59 ± 4.94	78	9.53 ± 5.61	108
Sandra et al. [47] *	2.9 ± 1.2	37	2.47 ± 1.3	38
Yusufu et al. [44] *	12.1 ± 5.3	30	13.2 ± 5.7	30
Zeng et al. [48] *	9.73 ± 5.91	182	9.97 ± 5.19	102

Studies marked with an asterisk (*) reported non-significant differences between T2DM and control groups (*p* > 0.05).

**Table 4:** Schirmer’s test values in diabetic and non-diabetic groups.

Study	T2DM (mm)	Number of Subjects	Control (mm)	Number of Subjects
Fan et al. [51]	4.82 ± 5.86	167	10.00 ± 7.78	68
Kesarwani et al. [39]	9.73 ± 4.97	80	25.84 ± 7.32	50
Manchikanti et al. [54]	9.57 ± 9.33	21	22.57 ± 6.79	21
Qu et al. [41]	6.76 ± 4.33	87	13.78 ± 2.26	51
Wu et al. [57]	9.59 ± 3.17	99	13.58 ± 2.92	40
Yang et al. [43]	8.68 ± 3.79	64	12.26 ± 4.49	64
Yang et al. [60]	7.82 ± 3.26	48	14.0 ± 5.98	16
Zeng et al. [48]	6.76 ± 5.76	182	9.25 ± 8.07	102
Zhang et al. [49]	5.7 ± 1.6	60	8.4 ± 2.4	60
Zhmud et al. [58]	6.32 ± 2.60	34	10.12 ± 2.49	26
Cakir et al. [37] *	13.63 ± 5.63	80	14.00 ± 5.73	20
Liang et al. [53] *	8.17 ± 6.72	76	9.26 ± 5.56	183
Lin et al. [40] *	5.57 ± 4.70	78	6.55 ± 5.93	108
Liu et al. [45] *	8.21 ± 7.16	56	10.1 ± 6.0	29
Lyu et al. [46] *	9.50 ± 7.00	87	10.25 ± 7.25	49
Stuard et al. [42] *	19.1 ± 8.21	18	17.8 ± 7.9	22
Tóth et al. [55] *	10.9 ± 10.7	44	8.8 ± 6.2	39
Yusufu et al. [44] *	12.4 ± 6.0	30	12.6 ± 6.5	30
Zou et al. [50] *	11.35 ± 4.77	20	13.90 ± 6.17	10

Studies marked with an asterisk (*) reported non-significant differences between T2DM and control groups (*p* > 0.05).

**Table 5:** OSDI scores in diabetic and non-diabetic groups.

Study	T2DM	Number of Subjects	Control	Number of Subjects
Han et al. [52]	19.81± 2.45	197	4.00 ± 1.44	60
Liu et al. [45]	16.23 ± 13.87	56	10.0 ± 6.1	29
Manchikanti et al. [54]	42.95 ±17.38	21	16.75 ± 5.45	21
Qu et al. [41]	30.90 ± 15.76	87	3.84 ± 6.90	51
Sandra et al. [47]	22.2 ± 7.93	37	16.2 ± 10.60	38
Stuard et al. [42]	19.08 ± 14.28	18	17.75 ± 16.15	22
Trindade et al. [56]	21.99 ±21.37	37	9.19 ± 11.71	23
Wu et al. [57]	19.98 ± 8.91	99	10.31 ± 1.45	40
Yang et al. [60]	12.71 ± 10.45	48	11.21 ± 8.21	16
Yu et al. [38]	23.02 ±13.13	118	12.11 ±6.48	100
Yusufu et al. [44]	9.2 ± 11.0	30	8.9 ± 12.9	30
Zhang et al. [49]	6.4 ± 1.7	60	4.4 ± 1.2	60
Zhmud et al. [58]	19 ± 4.0	34	13 ± 3.0	26
Liang et al. [53] *	21.88 ±12.73	76	20.19 ± 11.12	183
Lin et al. [40] *	12.79 ± 13.91	78	13.55 ± 16.42	108

Studies marked with an asterisk (*) reported non-significant differences between T2DM and control groups (*p* > 0.05).

**Table 6:** TMH in diabetic and non-diabetic groups.

Study	T2DM TMH (μm)	Number of Subjects	Control TMH (μm)	Number of Subjects
Fan et al. [51]	0.35 ± 0.12	167	0.40 ± 0.12	68
Han et al. [52]	0.22 ± 0.33	197	0.28 ± 0.12	60
Lin et al. [40]	0.19 ± 0.10	78	0.22 ± 0.14	108
Qu et al. [41]	0.22 ± 0.05	87	0.27 ± 0.11	51
Trindade et al. [56]	0.25 ± 0.08	37	0.30 ± 0.08	23
Yang et al. [60]	0.24 ± 0.06	48	0.27 ± 0.07	16
Zhang et al. [49]	0.17 ± 0.02	60	0.19 ± 0.01	60
Liang et al. [53] *	0.13 ± 0.03	76	0.13 ± 0.03	183
Lyu et al. [46] *	0.2 ± 0.07	87	0.20 ± 0.09	49
Sandra et al. [47] *	0.20 ± 0.08	37	0.21 ± 0.07	38
Yusufu et al. [44] *	0.31 ± 0.12	30	0.31 ± 0.12	30
Zeng et al. [48] *	0.23 ± 0.59	182	0.22 ± 0.86	102

Studies marked with an asterisk (*) reported non-significant differences between T2DM and control groups (*p* > 0.05).

**Table 7:** Risk of bias assessment for included cross-sectional studies.

Study	D1	D2	D3	Overall
Fan et al. [51]	Moderate	Moderate	Low	Low/Moderate
Han et al. [52]	Moderate	Moderate	Low	Low/Moderate
Kesarwani et al. [39]	Moderate	Moderate	Low	Low/Moderate
Liang et al. [53]	Moderate	Moderate	Low	Low/Moderate
Lin et al. [40]	Moderate/High	Moderate	Low	Moderate
Liu et al. [45]	Low	Low	Low/Moderate	Low
Lyu et al. [46]	Moderate	Moderate	Low	Low/Moderate
Manchikanti et al. [54]	Moderate/High	Moderate	Low	Moderate
Mangoli et al. [59]	Moderate	Moderate	Low/Moderate	Low/Moderate
Qu et al. [41]	Low	Low	Low	Low
Sandra et al. [47]	Moderate	Moderate	Low	Low/Moderate
Stuard et al. [42]	Moderate	Moderate	Low	Low/Moderate
Tóth et al. [55]	Moderate	Moderate	Low	Low/Moderate
Trindade et al. [56]	Moderate	Moderate	Low	Low/Moderate
Wu et al. [57]	Low/Moderate	Moderate	Low	Low/Moderate
Yang et al. [43]	Low/Moderate	Moderate	Low	Low/Moderate
Yu et al. [38]	Moderate	Moderate	Low	Low/Moderate
Zeng et al. [48]	Low	Moderate	Low	Low
Zhang et al. [49]	Low	Moderate	Low	Low
Zhmud et al. [58]	High	Moderate	Low	Moderate
Zou et al. [50]	High	Moderate	Low	Moderate

Domains: D1—Bias due to selection of participants; D2—Bias in measurement of exposure and outcomes; D3—Bias in control of confounding. Overall judgment is presented for each study.

## Data Availability

No new data was created during this study. Data extracted from the included studies is available from the corresponding author on reasonable request.

## Supplementary Materials

The following supporting information can be downloaded at https://www.mdpi.com/article/10.3390/diagnostics15243104/s1; Table S1: Boolean search strategies used across databases.
