# Supplementary material for: Tear Film Alterations in Type 2 Diabetes Mellitus: A Systematic Review and Meta-Analysis
Source: Diagnostics (Basel). 2025 Dec 6;15(24):3104. doi: 10.3390/diagnostics15243104 (PMC12732288; doi:10.3390/diagnostics15243104)
Supplement: Supplementary file 1 [file diagnostics-15-03104-s001.zip › diagnostics-3981671-supplementary.pdf]

diagnostics

MDPI

Supplementary Material

Tear Film Alterations in Type 2 Diabetes Mellitus: A System-atic Review and Meta-Analysis

Delius Mario Ghenciu, Alexandra Ioana Dănilă, Emil Robert Stoicescu, Adrian Neagu and Laura Andreea Ghen-ciu

Table S1. Boolean search strategies used across databases

Database	Search Strategy
**PubMed**	("diabetes mellitus"[MeSH Terms] OR "diabetes mellitus" OR "type 2 diabetes") AND ("tear film" OR "dry eye" OR "non-invasive tear break-up time" OR "NIBUT" OR "invasive tear break-up time" OR "IBUT" OR "tear meniscus height" OR "OSDI" OR "Ocular Surface Disease Index" OR "Schirmer test")
**Google Scholar**	("diabetes mellitus" OR "type 2 diabetes") AND (intitle:"tear film" OR intitle:"dry eye" OR intitle:"Ocular Surface Disease Index" OR intitle:OSDI)
**Web of Science**	("diabetes mellitus" OR "type 2 diabetes") AND ("tear film" OR "dry eye" OR "non-invasive tear break-up time" OR NIBUT OR "invasive tear break-up time" OR IBUT OR "tear meniscus height" OR OSDI OR "Oc-ular Surface Disease Index" OR "Schirmer test")
**Scopus**	TITLE-ABS-KEY (("diabetes mellitus" OR "type 2 diabetes" OR "type II diabetes" OR T2DM) AND ("tear film" OR "dry eye" OR "non-invasive tear break-up time" OR "noninvasive tear break-up time" OR "tear break-up time" OR "tear breakup time" OR "tear break up time" OR NIBUT OR IBUT OR TBUT OR NIKBUT OR "tear meniscus height" OR TMH OR OSDI OR "Ocular Surface Disease Index" OR "Schirmer test" OR "Schirmer's test")) AND PUBYEAR > 2015 AND PUBYEAR < 2026
